# Acute kidney injury, plasma lactate concentrations and lactic acidosis in metformin users: A GoDarts study

**DOI:** 10.1111/dom.12978

**Published:** 2017-07-05

**Authors:** Paul J. Connelly, Mike Lonergan, Enrique Soto‐Pedre, Louise Donnelly, Kaixin Zhou, Ewan R. Pearson

**Affiliations:** ^1^ Division of Molecular and Clinical Medicine, School of Medicine University of Dundee Dundee UK

**Keywords:** acute kidney injury, database research, lactic acidosis, metformin, type 2 diabetes

## Abstract

**Aims:**

Metformin is renally excreted and has been associated with the development of lactic acidosis. Although current advice is to omit metformin during illnesses that may increase the risk of acute kidney injury (AKI), the evidence supporting this is lacking. We investigated the relationship between AKI, lactate concentrations and the risk of lactic acidosis in those exposed to metformin.

**Materials and Methods:**

We undertook a population‐based case‐control study of lactic acidosis in 1746 participants with Type 2 diabetes and 846 individuals without diabetes with clinically measured lactates with and without AKI between 1994 and 2014. AKI was stratified by severity according to “Kidney Disease: Improving Global Outcomes” guidelines. Mixed‐effects logistic and linear regression were used to analyse lactic acidosis risk and lactate concentrations, respectively.

**Results:**

Eighty‐two cases of lactic acidosis were identified. In Type 2 diabetes, those treated with metformin had a greater incidence of lactic acidosis [45.7 per 100 000 patient years; 95% confidence interval (CI) 35.9‐58.3] compared to those not exposed to this drug (11.8 per 100 000 patient years; 95% CI 4.9‐28.5). Lactate concentrations were 0.34 mmol/L higher in the metformin‐exposed cohort (P < .001). The risk of lactic acidosis was higher in metformin users [odds ratio (OR) 2.3; P = .002] and increased with AKI severity (stage 1: OR 3.0, P = .002; stage 2: OR 9.4, P < .001; stage 3: OR 16.1, P < .001).

**Conclusions:**

A clear association was found between metformin, lactate accumulation and the development of lactic acidosis. This relationship is strongest in those with AKI. These results provide robust evidence to support current recommendations to omit metformin in any illness that may precipitate AKI.

## 1 INTRODUCTION

Metformin is the first‐line treatment of Type 2 diabetes recommended in both national and international guidelines. Metformin has an extremely favourable clinical profile: it is weight neutral, does not cause hypoglycaemia, and is superior to equivalent sulphonylurea or insulin therapy in the reduction of cardiovascular and all‐cause mortality.^1^ However, adverse reactions to this drug are common, with as many as 30% of metformin users developing gastrointestinal side‐effects.^2^

One risk associated with metformin use is the development of lactic acidosis, a potentially fatal condition. Lactic acidosis is defined as an elevated blood lactate concentration (>5 mmol/L) along with a decreased blood pH (<7.35) and an increased anion gap.^3^ The exact mechanism responsible for this condition is not fully understood; however, it is likely a consequence of metformin inhibition of the mitochondrial respiratory chain, thereby promoting anaerobic metabolism and lactate accumulation.^4^ This condition has a mortality of up to 50% per episode,^5^ and is believed to occur at a rate of about 1 per 23 000 to 30 000 person‐years of metformin use.^6^

Epidemiological studies have investigated the factors contributing to the development of lactic acidosis associated with metformin use.^3^, ^7^, ^8^, ^9^, ^10^ As metformin is exclusively excreted renally, the emphasis of these studies has been the role of chronic kidney disease (CKD) in the development of lactic acidosis.^8^, ^9^ Due to conflicting results there is not yet a consensus on their interpretation, which has led to the suggestion that it may be appropriate to relax current metformin‐prescribing practices in those with chronic renal impairment.^6^, ^11^ Nonetheless, the potential risk of metformin in severe renal disease has again been recently highlighted.^12^

There is, however, limited evidence of a link between metformin‐induced lactic acidosis and alterations in metformin pharmacokinetics. Case reports of acute metformin overdoses have shown that sudden increases in plasma metformin concentrations precede the development of lactic acidosis.^13^, ^14^ Dehydration and acute kidney injury (AKI) have been cited as precipitating factors in the development of lactic acidosis in those established on stable metformin therapy.^15^, ^16^, ^17^, ^18^ The current advice advocated by the UK Renal Association and NHS England, as part of the “Think Kidneys” initiative (http://www.thinkkidneys.nhs.uk), and by a number of other guidelines, is that metformin should be omitted during illness that may predispose to AKI, such as diarrhoea and vomiting.^4^ However, the evidence available concerning the impact of acute derangements in renal function upon the risk of precipitating metformin‐associated lactic acidosis is limited to small case series and has not been the subject of any population‐based epidemiological analysis. Consequently, we used a large population cohort, for whom biochemistry and prescribing data were available, to investigate the effect of AKI on plasma lactate concentrations and lactic acidosis in users and non‐users of metformin. In addition, AKI was stratified according to severity (stages 1‐3) in order to better characterise any observed relationship.

## 2 MATERIALS AND METHODS

### 2.1 Study setting and design

A population‐based case‐control study of lactic acidosis was conducted in Tayside, UK. Using the Genetics of Diabetes Audit and Research Tayside (GoDARTs) cohort, we identified a base population of approximately 18 000 individuals.^18^ Participants were eligible for inclusion if they were over 18 years old with no history of Type 1 diabetes.

GoDARTs participants with a measured lactate concentration were included in the source population of this study. Lactate concentrations were measured on clinical grounds with no fixed duration between measurements. This population was observed between January 1, 1994 and March 1, 2014. All lactic acidosis events, defined as a lactate > 5 mmol/L coincident with bicarbonate <18 mmol/L, were identified retrospectively from this database. These cases were compared with the remaining source population who had at least one lactate measure but no incident lactic acidosis. For each lactate measure, individuals were defined as being metformin treated if they encashed a metformin prescription within the 3 months before the lactate measure. Within the population of participants without diabetes, no metformin prescriptions were observed.

Participants with AKI were identified and stratified by severity according to the current “Kidney Disease: Improving Global Outcomes (KDIGO)” guidelines, which are based on serum creatinine.^19^ The urine output criterion for the diagnosis of AKI was omitted. Stage 1 AKI was defined as a creatinine rise of ≥26.5 µmol/L within 48 hours or a creatinine 1.5 to 1.9 times the baseline reference value. Stage 2 AKI was defined as 2.0 to 2.9 times increase in baseline creatinine. Stage 3 AKI was defined as ≥3.0 times baseline creatinine increase or increases of ≥354 µmol/L. Patients were coded as 1 to 3 based upon AKI staging, and 0 (AKI stage 0) in the absence of AKI. In accordance with the UK Renal Association guidance, the reference creatinine was defined as the lowest creatinine value recorded within 3 months before the event or estimated from the nadir creatinine following AKI recovery.^20^, ^21^, ^22^ Patients were also grouped into categories by baseline estimated glomerular filtration rate (eGFR) ranges ≥60 mL/min/1.73 m^2^ (CKD stages 1 and 2), eGFR ranges 30 to 59 mL/min/1.73 m^2^ (CKD stages 3A and 3B), and eGFR ranges <30 mL/min/1.73 m^2^ (CKD stages 4 and 5).^19^

### 2.2 Statistical analysis

Baseline participant characteristics of study participants were collected (Table 1) and compared using anova for continuous measures and chi‐squared test for categorical measures. A Cox proportional hazard model was used to investigate mortality following the first lactate measurement. Crude incidence rates for participants without diabetes and with Type 2 diabetes (metformin users and non‐users) were calculated along with 95% confidence intervals (95% CI) for the upper and lower limits based on the Poisson distribution. The base GoDARTs population was used as the denominator for this measure. Exposure was calculated from January 1, 1994 to the end of the study, the date of the first episode of lactic acidosis or the date of death. Data for each individual were censored following either of these events. During this observational period, patients encashing at least one metformin prescription were considered “metformin‐exposed.” Fisher's exact test was used to calculate differences in incidence rates.

**Table 1:** Baseline characteristics of study patients

	Non‐diabetic cohort	Type 2 diabetes cohort	
		Non‐users of metformin	Users of metformin
*n*	846	709	1037
Age (years)	74.5 (12.2)	74.4 (10.6)	70.9 (11.1)
Gender, male	56.4%	52.1%	55.9%
Deaths during study period, *n* (% of total deaths)	319 (37.6%)	384 (54.2%)	327 (31.5%)
Lactic acidosis cases, *n* (% of total of lactic acidosis cases)	12 (14.6%)	28 (34.1%)	42 (51.2%)
Lactate (mmol/L)	1.9 (1.8)	2.4 (2.0)	2.8 (2.2)
AKI stages, *n* (% of cohort with each stage of AKI)			
Stage 0	602 (71.1%)	350 (49.4%)	639 (61.6%)
Stage 1	137 (16.2%)	172 (24.3%)	219 (21.2%)
Stage 2	62 (7.3%)	107 (15.1%)	117 (11.3%)
Stage 3	45 (5.3%)	80 (11.3%)	62 (5.9%)
CKD stage, *n* (% of cohort with each stage of CKD)			
Stage 1	434 (51%)	242 (34.1%)	512 (49.4%)
Stage 2	283 (33.4%)	210 (29.6%)	360 (34.7%)
Stage 3A	86 (10.2%)	110 (15.5%)	126 (12.1%)
Stage 3B	35 (4.1%)	98 (13.8%)	32 (3.1%)
Stage 4	8 (0.9%)	47(6.6%)	7 (0.7%)
Stage 5	0 (0%)	2 (0.3%)	0 (0%)
Median CRP (mmol/L)	73 (114.3)	72.5 (121.0)	62 (120.3)
HBA1c (%)	N/A	8.1 (1.9)	7.9 (1.8)

Abbreviations: AKI, acute kidney injury; CKD, chronic kidney disease; CRP, C‐reactive protein.

Data expressed as mean (SD) unless otherwise stated.

The primary outcome, lactic acidosis, was analysed using a mixed‐effects logistic regression analysis. A mixed linear regression model was then fitted to assess the effect of selected covariates upon the continuous variable, lactate (mmol/L). Mixed‐effects models are used for grouped or clustered data where observations within a cluster cannot be assumed to be independent. These models include both fixed‐effects, to estimate average outcomes across the population, and random‐effects, that represent variation between the subgroups of data points. Random effects were included when the intra‐class correlation coefficient ≥0.1.^23^ Likelihood ratio testing was used to confirm the suitability of mixed‐effects models compared with standard regression.

A backwards‐stepwise method was used for model selection. Covariate terms were excluded if they were found to be non‐significant within the model (*P* > .05). The covariates selected as candidate terms for inclusion in the models were the categorical variables metformin use, gender, AKI stage and CKD stage, and the continuous variables C‐reactive protein (CRP; mmol/L) and age at point of lactate measurement. CRP was included as a covariate as a measure of infection or inflammation that might have precipitated the lactate measurement. The last recorded HbA1c (Diabetes Control and Complications Trial %) was used in the analysis of data from only patients with diabetes. Metformin was treated as a random coefficient within the mixed models permitting alternating uses throughout the study period. An interaction term between AKI stage and the use of metformin was also incorporated into a mixed linear regression analysis of lactate concentrations in the cohort of patients with Type 2 diabetes.

## 3 RESULTS

### 3.1 Study cohort and participant characteristics

Lactate measurements were available for 1746 patients with Type 2 diabetes and 846 people without diabetes (Table 1). One‐hundred and fourteen individuals (10.1% of metformin users) had lactate levels recorded both during periods of metformin use and non‐use. In this sub‐cohort, mean lactate measurements were 2.14 mmol/L when not exposed to metformin and 2.67 mmol/L when exposed to this drug (mean difference 0.52 mmol/L; 95% CI 0.17‐0.89; *P* = .004).

The mean number of measurements per person within each group was 1.9 (SD 1.6), 2.3 (SD 2.2) and 2.4 (SD 2.3) in the non‐diabetic group, non‐metformin users and metformin users, respectively [Kruskal–Wallis *H*‐test; χ^2^(2) = 28.6; *P* = .0001]. Seven individuals in the metformin‐using group (0.8%) had been prescribed metformin despite having an eGFR <30 mL/min/1.73 m^2^.

In this study cohort who had lactate measured, metformin‐treated patients compared with non‐metformin‐treated patients with Type 2 diabetes were younger (*P* < .001), with less overall AKI (*P* < .001) and less CKD (*P* < .001), and had lower subsequent deaths in the time after their first lactate measure [hazard ratio (HR) 0.51; *P* < .001; 95% CI 0.43‐0.59]. These results suggest that metformin‐treated patients who had lactate measured had generally better health at the time of blood sampling compared with non‐metformin‐treated patients, yet their serum lactate was increased (*P* < .001). Overall, the median CRP ranged from 62 to 73 mmol/L, which suggests that lactate concentrations were measured during periods of acute illness and likely sepsis.

### 3.2 Lactic acidosis patient characteristics

Eighty‐two individuals who developed lactic acidosis were identified (Table 2). AKI was present in 79.3% of cases with lactic acidosis (stage 1: 19.5%; stage 2: 30.5%; stage 3: 29.3%). The crude incidence of lactic acidosis was 24.3 cases per 100 000 patient years (95% CI 19.5‐30.1). Crude incidence rates were higher within the Type 2 diabetes cohort (37.9 per 100 000 patient years; 95% CI 30.0‐48.0) compared with those without diabetes (7.8 per 100 000 patient years; 95% CI 4.4‐13.7; *P* < .001). Within the Type 2 diabetes cohort, those exposed to metformin had an incidence of 45.7 per 100 000 patient years (95% CI 35.9‐58.3) compared with 11.8 per 100 000 patient years in patients never exposed to metformin (95% CI 4.9‐28.5, *P* < .001).

**Table 2:** Clinical and biochemical characteristics of patients with incident cases of lactic acidosis (cases) and the remaining study population (non‐cases)

	Lactic acidosis cases	Non‐cases
*n*	82	2510
Age (years)	73.5 (9.5)	73.0 (11.5)
Male (%)	65.8%	54.6%
Lactate (mmol/L)	9.3 (3.5)	2.3 (1.7)
Bicarbonate (mmol/L)	11.9 (3.9)	23.5 (5.0)
Type 2 Diabetes, *n* (% of cohort)	70 (85.4%)	1676 (66.8%)
Users of metformin	42 (60%)	970 (57.9%)
Non‐users of metformin	28 (40%)	706 (42.1%)
AKI stages, *n* (% of cohort with each stage of AKI)		
Stage 0	17 (20.7%)	1569 (62.5%)
Stage 1	16 (19.5%)	508 (20.2%)
Stage 2	25 (30.5%)	266 (10.6)
Stage 3	24 (29.3%)	167 (6.6%)
Baseline eGFR, *n* (% of cohort)		
>60 mL/min/1.73 m^2^	59 (71.9%)	1979 (78.8%)
30‐59 mL/min/1.73 m^2^	19 (23.2%)	468 (18.6%)
<30 mL/min/1.73 m^2^	4 (4.9%)	63 (2.5%)

Abbreviations: AKI, acute kidney injury; eGFR, estimated glomerular filtration rate.

Data expressed as mean (SD) unless otherwise stated.

### 3.3 Lactic acidosis mixed‐effects logistic regression

A random intercept mixed logistic regression analysis was used to calculate the odds ratios (ORs) for the development of lactic acidosis (Table 3). Univariate analyses showed associations between lactic acidosis and metformin use (OR 1.8; *P* = .011), Type 2 diabetes (OR 2.3; *P* = .004) and AKI (stage 1: OR 3.2; stage 2: 9.5; stage 3: 15.9; *P* < .002). CKD was not significantly associated with the development of this condition (CKD stage 4/5; OR 2.8; *P* = .068); however, only five instances of lactic acidosis occurred in patients with CKD stage 4/5, and only one was prescribed metformin.

**Table 3:** Mixed‐effects logistic regression analyses assessing the risk of lactic acidosis

	Covariate	OR	*P*‐value	95% CI
Univariate analysis				
	AKI Stage			
	1	3.2	.001	1.6‐6.6
	2	9.6	<.001	4.7‐19.3
	3	15.9	<.001	7.7‐32.8
	Metformin	1.8	.011	1.1‐2.9
	Type 2 Diabetes	2.3	.018	1.2‐4.8
Multivariate analysis				
	AKI Stage			
	1	3.0	.002	1.5‐6.1
	2	9.4	<.001	4.7‐18.8
	3	16.1	<.001	7.9‐33.0
	CKD Stage 4/5	3.6	.028	1.1‐11.6
	Metformin	2.3	.002	1.4‐3.8

Abbreviations: AKI, acute kidney injury; CI, confidence interval; OR, odds ratio.

Multivariate analysis including both those with and without Type 2 diabetes demonstrated that metformin use (OR 2.3; *P* = .002; 95% CI 1.4‐3.8) increased the likelihood of developing lactic acidosis (Table 3). There was also a marked association between the development of lactic acidosis and AKI severity. Later stages of CKD were also associated with lactic acidosis. The presence of Type 2 diabetes was not found to have a significant effect when included in this multivariate model (*P* = .26). When diabetes status was included into this model; AKI, CKD stage 4/5 and metformin exposure continued to be associated with the development of lactic acidosis.

A sub‐cohort analysis was then performed in only those prescribed metformin. Only one patient with CKD stages 4/5 developed lactic acidosis. We therefore combined this group with those with CKD stages 3A/3B to compare those eGFR > 60 mL/min/1.73 m^2^ with those with chronic impairment. A multivariate analysis demonstrated that AKI stage 1 (OR = 3.8; *P* = .009; 95% CI 1.4‐10.4), stage 2 (OR 10.5; *P* < .001; 95% CI 3.6‐30.8), stage 3 (OR 24.6; *P* < .001; 95% CI 7.9‐75.9), CKD stages with eGFR < 60 mL/min/1.73 m^2^ (OR 2.7; *P* = .021; 95% CI 1.2‐6.5) and HbA1c (OR 1.2; *P* = .02; 95% CI 1.0‐1.4) were associated with the development of lactic acidosis.

### 3.4 Sensitivity analysis

In a sensitivity analysis, lactic acidosis risk was assessed using a non‐mixed‐effects multivariate logistic regression model. This analysis yielded similar results to the mixed‐effects model outlined in Table 3. In this sensitivity analysis, AKI (stage 1: OR 2.8; stage 2: OR 7.7; stage 3: OR 11.1; *P* = .005) and metformin use (OR 1.9; *P* = .002) were associated with lactic acidosis; however, CKD stage 4/5 was not found to be statistically significant in this model.

### 3.5 Plasma lactate concentrations

**Figure 1:**
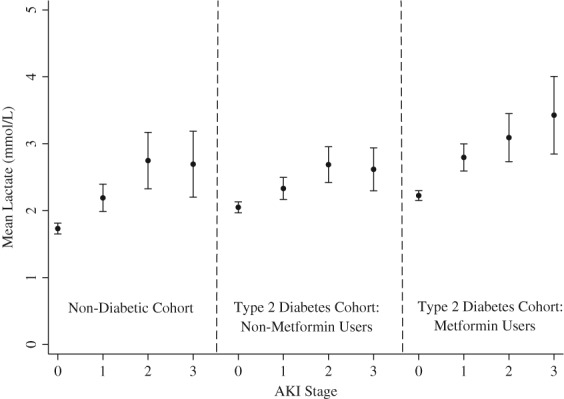
Mean lactate concentrations with 95% confidence intervals (CI). Participants were separated into non‐diabetic groups (left), and patients with Type 2 diabetes who were not prescribed metformin (centre) and those who were metformin users (right). Mean lactate concentrations were derived from the all lactate measurements made during the study period within each group

In univariate analyses, metformin use (*B* = 0.39 mmol/L; *P* < .001), AKI (stage 1: *B* = 0.49 mmol/L; stage 2: *B* = 0.89 mmol/L: stage 3: *B* = 0.94 mmol/L; *P* < .001), male gender (*B* = 0.19 mmol/L; *P* = .001), Type 2 diabetes (*B* = 0.42 mmol/L; *P* < .001) and CRP (*B* = 0.001 mmol/L; *P* < .001) were associated with higher lactate concentrations (Table 4). No effects of CKD stage were detected (*P* = .875). Lactate concentrations in the non‐diabetic group, metformin users and non‐metformin users with Type 2 diabetes at varying stages of AKI are shown in Figure 1.

**Table 4:** Lactate concentration mixed‐effects analyses

	Covariate	Coefficient (*B*)	*P*‐value	95% CI
Univariate analysis				
	AKI Stage			
	1	0.49	<.001	0.38‐.61
	2	0.89	<.001	0.74‐1.04
	3	0.94	<.001	0.75‐1.13
	Metformin	0.39	<.001	0.28‐0.51
	Type 2 diabetes	0.42	<.001	1.34‐1.76
	Male gender	0.19	.001	1.76‐2.13
	CRP (mmol/L)	0.001	<.001	0.0003‐0.001
Multivariate analysis				
	AKI Stage			
	1	0.48	<.001	0.36‐0.60
	2	0.89	<.001	0.73–1.03
	3	0.92	<.001	0.74‐1.11
	Metformin	0.34	<.001	0.21‐0.47
	Male gender	0.19	.001	0.09‐0.31
	Type 2 diabetes	0.19	.006	0.05‐0.32
Multivariate analysis (Type 2 diabetes cohort only)				
	AKI Stage			
	1	0.31	.002	0.11‐0.51
	2	0.67	<.001	0.43‐0.90
	3	0.57	<.001	0.29‐0.85
	Metformin	0.16	.053	−0.002‐0.33
	AKI*Metformin			
	1	0.33	.025	0.04‐0.62
	2	0.37	.041	0.01‐0.73
	3	0.82	.001	0.38‐1.26
	HbA1C (%)	0.04	.021	0.01‐0.07

AKI, acute kidney injury; CI, confidence interval.

In a multivariate analysis (Table 4), compared with patients without AKI, lactate measurements increased in patients with stage 1 (*B* = 0.48 mmol/L; *P* < .001), stage 2 (*B* = 0.89 mmol/L; *P* < .001) and stage 3 AKI (*B* = 0.92 mmol/L; *P* < .001). Additional significant covariates included metformin use (*P* < .001), male gender (*P* = .001) and Type 2 diabetes (*P* < .001). In those with type 2 diabetes, inclusion of an interaction term in the model for lactate concentrations showed an interaction between metformin exposure and AKI status (Table 4). The effect of stage 3 AKI upon lactate concentrations was higher in those using metformin, where the lactate measure was 0.82 mmol/L greater than those not taking metformin. Interestingly, in the absence of AKI, metformin was not found to be statistically significant in this model (*P* = .053), thereby highlighting the importance of deranged renal function upon metformin's capacity to increase lactate concentrations.

A multivariate analysis was then performed stratified by renal function. In those with “normal” renal function (eGFR >60 mL/min/1.73 m^2^), lactate concentrations were increased in patients with stage 1 (*B* = 0.49 mmol/L; *P* < .001), stage 2 (*B* = 1.07 mmol/L; *P* < .001) and stage 3 AKI (*B* = 1.04 mmol/L; *P* < .001). Lactate concentrations were also independently associated with metformin use (*B* = 0.26 mmol/L; *P* = .001), Type 2 diabetes (*B* = 0.22 mmol/L; *P* = .005) and male gender (*B* = 0.2 mmol/L; *P* = .001). In those with eGFR≤60 mL/min/1.73 m^2^, only AKI (stage 1: *B* = 0.45 mmol/L; stage 2: *B* = 0.31 mmol/L; stage 3: *B* = 0.57 mmol/L; *P* < .04) and metformin use (*B* = 0.57 mmol/L; *P* < .001) were associated with increased lactate concentrations.

Lastly, an analysis was performed in metformin users only. A multivariate analysis showed that AKI stage 1 (*B* = 0.4 mmol/L; *P* < .001; 95% CI 0.4‐0.8), stage 2 (*B* = 1.02 mmol/L; *P* < .001; 95% CI 0.7‐1.3), stage 3 (*B* = 1.36 mmol/L; *P* < .0001; 95% CI 1.02‐1.71), CKD stages 4 and 5 (*B* = 1.8 mmol/L; *P* = .005; 95% CI 0.55‐3.1) and HbA1c (*B* = 0.05 mmol/L; *P* = .045; 95% CI 0.001‐0.1) were associated with higher lactate concentrations. In those with an eGFR >60 mL/min/1.73 m^2^, AKI was the only significant covariate (stage 1: *B* = 0.66 mmol/L; stage 2: *B* = 1.09 mmol/L; stage 3: *B* = 1.19 mmol/L; *P* < .001). In participants with an eGFR < 60 mL/min/1.73 m^2^, only severe AKI (stage 3) was found to be significant (*B* = 2.0 mmol/L; *P* < .001).

## 4 DISCUSSION

We have undertaken a large population‐based cohort study to assess the association between metformin, AKI, and the risk of developing lactic acidosis and high lactate concentrations. We have shown that metformin‐treated individuals are more than twice as likely to develop this condition when compared with non‐metformin‐treated individuals with or without diabetes. The impact of metformin upon lactate concentrations is evident regardless of baseline renal function; however, approximately 80% of lactic acidosis cases identified within this study demonstrated evidence of AKI. Furthermore, the presence of AKI increases the risk of lactic acidosis in a stepwise fashion with similar trends identified when assessing lactate concentrations. An interaction between metformin and AKI was also demonstrated, suggesting the significance of acute derangements in renal function in the context of metformin exposure. Guidelines recommend that metformin should be withheld in the presence of AKI, or where patients are believed to be at risk of compromised renal function. However, the evidence supporting this practice has been lacking. Our data provide strong evidence of this presumed association, and highlight the significance of AKI severity in the accumulation of serum lactate and the risk of lactic acidosis.

The crude incidence rate for lactic acidosis of 45.7 per 100 000 patient years in those exposed to metformin during the study period is higher than those reported in recent studies, though similar rates do appear in the literature.^8^, ^9^, ^24^, ^25^ In 2010, Salpeter et al. published a Cochrane review comprising data from 347 comparative studies amounting to 70 490 patient years, which did not identify a single case of lactic acidosis.^7^ However, a significant proportion of studies excluded participants with renal dysfunction or conditions associated with lactic acidosis, such as hepatic or cardiovascular dysfunction. It is therefore unsurprising that no cases of this rare condition were identified in this low‐risk, well‐monitored population. A major strength of our study is the utilisation of a population‐based database, which is reflective of daily clinical practice with complete capture of all biochemistry in the population. Our high estimates may be a consequence of our biochemical diagnosis of this condition from direct assessment of laboratory investigations.

Previous studies have relied upon the interpretation of General Practice “read codes” for the diagnosis of lactic acidosis, which may introduce problems with diagnostic misclassification.^8^ Of the 82 cases that we biochemically identified, only 9 were found to have an ICD discharge code of acidosis, and 2 further patients had a cause of death coded as acidosis. The acidosis code includes “acidosis NOS,” “lactic acidosis,” “metabolic acidosis” and “respiratory acidosis.” There were 111 patients with acidosis codes that were not identified as having lactic acidosis in our study. This suggests that ICD codes are neither sensitive nor specific for identifying cases of lactic acidosis.

We show that within the population assessed, the use of metformin increases plasma lactate concentrations by 0.34 mmol/L on average, which is consistent with a previous study on lactate concentrations in 1024 individuals with normal renal function.^26^ In addition, we demonstrate that metformin is associated with an increased risk of lactic acidosis, with those prescribed metformin found to be more than twice as likely to receive a biochemical diagnosis of lactic acidosis as both individuals without diabetes and individuals receiving other treatments for diabetes. These results are independent of other clinical parameters including CKD and AKI. Our results also suggest that the effect of metformin may be independent of diabetes status. However, this conclusion is limited by the absence of metformin exposure in the non‐diabetic cohort.

The largest risk factor for elevated lactate concentrations and lactic acidosis in our study is the presence of AKI. Approximately 80% of lactic acidosis cases in this study presented with evidence of AKI. This is seen in individuals without diabetes and participants with Type 2 diabetes with or without metformin treatment. There is an interaction between metformin use and AKI stage, with the largest effect on lactate seen in those with AKI stage 3 who are also treated with metformin. This interaction has been previously reported in a lactic acidosis case‐control study.^27^ Patients with Type 2 diabetes have increased risk of AKI even following adjustments for risk factors such as chronic renal impairment, and are therefore an “at‐risk population.”^28^ A previous retrospective case series of 66 patients with metformin‐associated lactic acidosis demonstrated evidence of AKI in every patient.^16^ In that published cohort, metformin concentrations correlated well with both serum creatinine and plasma lactate levels. Careful consideration is therefore necessary before prescribing this medication during periods of impaired renal function, such as the development of gastroenteritis, sepsis or any intercurrent illness or procedures where dehydration may be likely, such as endoscopy.^15^

There is on‐going debate about the use of metformin in patients with CKD.^6^ Recently, two retrospective UK studies utilising the CPRD database have been performed. In one, an assessment of 223 968 metformin users and 34 571 patients with Type 2 diabetes naive to metformin reported a HR of 6.37 for lactic acidosis or elevated lactate concentration (>5 mmol/L) in metformin users with an eGFR <60 mL/min/1.73 m^2^.^8^ However, in the other, a retrospective analysis of 77 601 metformin‐using patients identified a total of 35 non‐fatal lactic acidosis events with no difference in incidence of lactic acidosis between groups stratified by renal function.^9^ In our study, we do show a threefold increase in the risk of lactic acidosis in those with CKD stage 4/5. However, there was limited use of metformin in this CKD group who had lactates measured, which reflects the common prescribing practices of metformin in those with chronic renal impairment. The association observed between CKD4/5 and high lactate concentrations and acidosis is likely driven by the non‐metformin users, and reflects an effect of chronic renal impairment *per se* rather than an interaction between metformin use and CKD.

There are a number of limitations to this study. Firstly, to be included in the source population a lactate measurement is required. The decision to measure lactate is likely to be biased, with a lower threshold for lactate to be measured in those who are metformin treated. This is reflected by the fact that metformin‐treated patients who have lactate measured are younger and have less co‐morbidities than non‐metformin‐treated patients. However, despite this selection bias, we show increased elevation of serum lactate concentrations and lactic acidosis risk in these patients. These data could also reflect that as the elevation of lactate is in part mediated by the direct effect of metformin, these patients do not need to be so sick to reach the biochemical threshold defining lactic acidosis. Secondly, due to the mortality associated with lactic acidosis, we cannot rule out the presence of survival bias. However, the impact of this is likely small, and such an effect would have led to underestimation of disease effect sizes. Thirdly, there were several potential confounders that could not be directly assessed, such as the nature of patient admissions, the use of intravenous contrast medium or the presence of contributing co‐morbidities, such as hepatic or cardiovascular disease. Fourthly, identification of lactic acidosis was reliant upon using bicarbonate as a biochemical marker of acidosis rather than direct estimates of arterial pH. Serum bicarbonate has, however, has been validated as an alternative predictive marker to arterial base deficit.^29^, ^30^ Finally, our available data did not allow direct assessment of metformin dose or plasma metformin concentrations, preventing the assessment of dose–response and pharmacokinetic relationships, respectively.^31^, ^32^

In this study, we utilise a population‐based cohort with complete capture of all incident lactate and bicarbonate measures rather than relying on low sensitivity and specificity “read” or ICD codes. We show that metformin use in patients with Type 2 diabetes increases plasma lactate concentrations and the risk of lactate acidosis, and that this association is particularly important in the context of AKI, and can be seen even in those with normal renal function. This study affirms and expands upon the available literature supporting this relationship, which until now has been limited to low levels of evidence. We believe that our results provide robust evidence to support the guidelines that patients should be aware of this potentially harmful side‐effect of metformin, and instructed to temporarily stop taking metformin during illnesses associated with acute renal impairment.

## ORCID

*Paul J. Connelly* http://orcid.org/0000-0002-1744-9977
